# Performance of ultrasound-guided attenuation parameter and 2D shear wave elastography in patients with metabolic dysfunction-associated steatotic liver disease

**DOI:** 10.1007/s00330-024-11076-w

**Published:** 2024-10-07

**Authors:** Roberto Cannella, Francesco Agnello, Giorgia Porrello, Alessandro Umberto Spinello, Giuseppe Infantino, Grazia Pennisi, Daniela Cabibi, Salvatore Petta, Tommaso Vincenzo Bartolotta

**Affiliations:** 1 Section of Radiology, Department of Biomedicine, Neuroscience and Advanced Diagnostics (BiND), University of Palermo, Via del Vespro 129, Palermo, 90127 Italy; 2 Section of Gastroenterology and Hepatology, Department of Health Promotion, Mother and Child Care, Internal Medicine and Medical Specialties (PROMISE), University of Palermo, Palermo, Italy; 3 Unit of Anatomic Pathology, Department of Health Promotion, Mother and Child Care, Internal Medicine and Medical Specialties (PROMISE), University of Palermo, Palermo, Italy

**Keywords:** Ultrasonography, Fatty liver, Nonalcoholic fatty liver disease, Fibrosis, Obesity

## Abstract

**Purpose:**

To assess the performance and the reproducibility of ultrasound-guided attenuation parameter (UGAP) and two-dimensional shear wave elastography (2D-SWE) in patients with biopsy-proven metabolic dysfunction-associated steatotic liver disease (MASLD).

**Methods:**

This study included consecutive adult patients with MASLD who underwent ultrasound with UGAP, 2D-SWE and percutaneous liver biopsy. The median values of 12 consecutive UGAP measurements were acquired by two independent radiologists (R1 and R2). Hepatic steatosis was graded by liver biopsy as: (0) < 5%; (1) 5–33%; (2) > 33–66%; (3) > 66%. Areas under the curve (AUCs) were calculated to determine the diagnostic performance. Inter- and intra-observer reliability was assessed with intraclass correlation coefficient (ICC).

**Results:**

A hundred patients (median age 55.0 years old) with MASLD were prospectively enrolled. At histopathology, 70 and 42 patients had grade ≥ 2 and 3 steatosis, respectively. Median UGAP was 0.78 dB/cm/MHz (IQR/Med: 5.55%). For the diagnosis of grade ≥ 2 steatosis, the AUCs of UGAP were 0.828 (95% CI: 0.739, 0.896) for R1 and 0.779 (95% CI: 0.685, 0.856) for R2. The inter- and intra-operator reliability of UGAP were excellent, with an ICC of 0.92 (95% CI: 0.87–0.95) and 0.95 (95% CI: 0.92–0.96), respectively. The median liver stiffness was 6.76 kPa (IQR/Med: 16.30%). For the diagnosis of advanced fibrosis, 2D-SWE had an AUC of 0.862 (95% CI: 0.757, 0.934), and the optimal cutoff value was > 6.75 kPa with a sensitivity of 80.6% and a specificity of 75.7%.

**Conclusion:**

UGAP and 2D-SWE provide a good performance for the staging of steatosis and fibrosis in patients with MASLD with an excellent intra-operator reliability of UGAP.

**Key Points:**

***Question*** *How well do ultrasound-guided attenuation parameter (UGAP) and two-dimensional shear wave elastography (2D-SWE) perform for quantifying hepatic steatosis and fibrosis?*

***Findings*** *UGAP had a maximum AUC of 0.828 for the diagnosis of grade ≥ 2 steatosis, and 2D-SWE had an AUC of 0.862 for diagnosing advanced fibrosis.*

***Clinical relevance*** *UGAP and 2D-SWE allow rapid, reproducible, and accurate quantification of hepatic steatosis and fibrosis that can be used for the noninvasive assessment of patients with metabolic dysfunction-associated steatotic liver disease.*

## Introduction

Nonalcoholic fatty liver disease (NAFLD), recently defined as metabolic dysfunction-associated steatotic liver disease (MASLD), is becoming the most common cause of chronic liver disease, with an estimated global prevalence of 30% in the general population, and it has been associated with metabolic dysfunction [1, 2]. MASLD can evolve into metabolic dysfunction-associated steatohepatitis (MASH), with hepatic fibrosis progressively leading to cirrhosis, with possible complications related to decompensated liver disease and the development of hepatocellular carcinoma. Liver fibrosis is considered the most important prognostic factor in patients with MASLD, and it has been associated with an increased risk of death [3]. Hepatic steatosis is the main criterion to define MASLD, and its quantification can be relevant for treatment monitoring [4]. Moreover, hepatic steatosis has been linked to an increased risk of cardiovascular events, diabetes mellitus and hypertension [4]. The actual reference standard for fibrosis staging and steatosis grading is liver biopsy, but this technique can be prone to sampling errors and complications, and its invasiveness prevents its use for close disease monitoring. Magnetic resonance elastography and MRI-PDFF can be considered the noninvasive gold standard for fibrosis and steatosis, respectively, but their scarce availability and high costs limit their use in clinical practice.

Ultrasound remains the primary imaging modality for the assessment of hepatic steatosis according to current guidelines [4, 5]. However, the sensitivity of B-mode ultrasound is suboptimal for the detection of mild hepatic steatosis (about 60% for non-quantitative assessment), it can be affected by the operator experience, and it has limited reliability [6–8]. Recently, ultrasound-based techniques have been developed to quantify hepatic steatosis through ultrasound bean attenuation measurement during B-mode examinations [9]. These techniques, such as ultrasound-guided attenuation parameter (UGAP), attenuation imaging (ATI) or tissue attenuation imaging (TAI^TM^), provide quantitative information related to the amount of hepatic steatosis and they have been integrated into conventional ultrasound equipment [8, 9]. Recent studies evaluated the performance of UGAP for the quantification of hepatic steatosis in patients with different etiologies of chronic liver disease with good-to-excellent performances [10–16]. Few studies assessed the performance of UGAP for the diagnosis of hepatic steatosis in patients with MASLD, using liver biopsy or controlled attenuation parameter (CAP) as reference standard [17–19]. Two-dimensional shear wave elastography (2D-SWE) has been extensively studied for the quantification of liver stiffness, and it can be acquired in the same ultrasound examination, providing relevant diagnostic implications for both steatosis and fibrosis assessment.

The primary purpose of this study is to assess the performance and reproducibility of ultrasound-guided attenuation parameter (UGAP) for the quantification of hepatic steatosis in patients with biopsy-proven nonalcoholic fatty liver disease. The secondary aim is to evaluate the performance of two-dimensional shear wave elastography (2D-SWE) for the assessment of hepatic fibrosis in patients with MASLD.

## Materials and methods

This prospective single-center study was approved by the Ethical Committee of our University Hospital (approval No. 01/2022). All participants provided written informed consent upon enrollment.

### Participants

Participants were enrolled between February 2022 and July 2023 according to the following inclusion criteria: (1) being older than 18 years; (2) new diagnosis of MASLD undergoing initial clinical evaluation in the absence of prior treatments; (3) underwent percutaneous liver biopsy according to clinical/biochemical and instrumental evaluation. The following exclusion criteria were considered: (1) history of other etiologies of chronic liver diseases, including viral hepatitis, alcohol consumption ≥ 30 g/day for men or ≥ 20 g/day for women, acute hepatitis, or hemochromatosis [4, 5]; (2) other causes of secondary hepatic steatosis, including use of hepatotoxic drugs that may cause steatosis; (3) aspartate transaminase (AST) and/or alanine transaminase (ALT) elevation > 5 times the normal limits.

All participants underwent clinical evaluation on the same day of the liver biopsy. The following data were collected: age, sex, body mass index (BMI), laboratory tests, and transient elastography (FibroScan®) with controlled attenuation parameter (CAP) measurements. Participants were graded as underweight (BMI < 18.5 kg/m^2^), normal weight (BMI from 18.5 to < 25 kg/m^2^), overweight (BMI from 25 to < 30 kg/m^2^), or obese (BMI ≥ 30 kg/m^2^) [4]. TE and CAP measurements were performed by trained operators who had previously performed at least 300 determinations in patients with chronic liver diseases. Measurements were acquired with FibroScan^®^ (Echosens), using the M and the XL probe when appropriate, after overnight fasting. Only patients with ten valid measurements and with reliable results according to published criteria were enrolled [20].

### Ultrasound measurements

Ultrasound examinations were performed with a dedicated ultrasound system (LOGIQ E10, GE Healthcare) equipped with a C1-6 convex probe. Ultrasound measurements were acquired by two abdominal radiologists (R1, R.C. with 8 years of experience, and R2, F.A. with 15 years of experience) who were blinded to the clinical and histopathological characteristics of the enrolled participants. The radiologists underwent a dedicated training session on UGAP measurements before beginning the study. The study protocol consisted of the following steps: (1) the first radiologist measured the skin-to-liver capsule distance and the splenic length, and acquired the UGAP and 2D-SWE measurements; (2) the second radiologist independently acquired the UGAP measurements to assess the inter-operator reproducibility; (3) the UGAP measurements were repeated a second time by the first radiologist to evaluate the intra-operator reproducibility.

All subjects enrolled were examined after fasting of 6 h minimum before UGAP, lying in a supine position with the right arm elevated above their head. The convex probe was placed on the right intercostal space along the mid-axillary line, in order to obtain ultrasound images of the right hepatic lobe. Measurements were obtained in breath hold. Images in B-mode and B-mode with color map overlay were simultaneously displayed during the examination. For UGAP sampling (GE Healthcare), a color map corresponding to the measurements with the highest accuracy is automatically provided by the software. The color map is placed at about 2 cm depth from the liver capsule. A fixed-size region of interest (ROI) is then placed in the homogeneous parts of the color map, carefully avoiding intrahepatic vessels, diaphragm, or rib shadow artifacts. A total of 12 UGAP measurements were acquired by each radiologist in different ultrasound frames. The attenuation coefficient measured in dB/cm/MHz was recorded for each measurement. The median (med) of the twelve measurements with its interquartile range (IQR) and IQR/Med ratio were provided. Measurements with IQR/Med equal or below 30% were considered reliable.

For 2D-SWE measurements (GE Healthcare), the 2D color and quality maps were placed in the right liver lobe at 1–2 cm depth from the liver capsule, without including intrahepatic vessels or artifacts. ROIs were placed in the color-coded map homogeneous areas. The attenuation coefficient measured in kPa was recorded for each measurement. The median (med) of twelve measurements with its interquartile range (IQR) and IQR/Med ratio were provided. Exams with twelve consecutive measurements with IQR/Med equal or below 30% were considered reliable.

### Reference standard

All percutaneous liver biopsies were obtained according to clinical indications in patients with suspected MASLD. The criteria for performing liver biopsy in patients with suspected MASLD were determined based on both clinical/biochemical assessment and instrumental evaluation. These criteria include the presence of hepatic steatosis on ultrasound (US) associated with at least one of the following parameters: (1) FIB-4 score ≥ 1.3 followed by further evaluation using transient elastography (FibroScan®) with a result ≥ 8 Kpa; (2) elevated AST and/or ALT and/or gamma-glutamyltransferase levels persisting for at least 6 months. The median interval time between liver biopsy and ultrasound measurements was 60 days. Liver biopsies were evaluated by a single pathologist (D.C. with more than 30 years of experience in liver pathology), blinded to the participant laboratory data and ultrasound measurements. Hepatic steatosis percentage was recorded and graded as grade 0 (if < 5%), grade 1 (5–33%), grade 2 (> 33–66%), and grade 3 (> 66%) [21]. The NAFLD activity score (NAS), lobular inflammation (0–3), and ballooning (0–2) were also provided for each patient. Hepatic fibrosis was defined according to the Kleiner score [21]. Patients with F2-F4 scores were defined as significant fibrosis, while patients with F3-F4 were defined as advanced fibrosis.

### Statistical analysis

Categorical variables are reported with numbers and percentages, and they were compared with the Pearson χ2 or Fisher exact test. Continuous variables are provided with median and IQR after testing with the Shapiro–Wilk normality test, and they were compared by using the Kruskal–Wallis or the Mann–Whitney *U* test. Correlation between UGAP measurements and other patients’ clinical characteristics was explored with the Spearman rank-order correlation coefficients. The intra- and inter-operator agreement of median UGAP measurements was evaluated using the intraclass correlation coefficient (ICC), with 95% confidence intervals (CIs), based on the absolute agreement with the 2-way mixed-effects model. Agreement was categorized as poor (ICC < 0.50), moderate (ICC between 0.50 and 0.75), good (ICC between 0.75 and 0.90), or excellent (ICC > 0.90) [22]. Bland-Altman plots were used to evaluate the magnitude of variation between intra- and inter-operator median UGAP measurements.

Area under the receiver operating characteristic curve (AUC) with its 95% confidence interval (95% CI) was calculated to assess the performance of UGAP for the diagnosis of grade ≥ 2 or grade 3 steatosis and the performance of 2D-SWE for the diagnosis of significant (F2-F4) and advanced (F3-F4) fibrosis. Optimal cutoffs with their sensitivity and specificity were determined based on the Youden index. AUCs were compared using the DeLong test. Univariate and multivariate binary logistic regression analyses were performed to assess variables associated with high UGAP values. The odds ratio (OR) with 95% CI was calculated.

A *p*-value < 0.05 was considered to be statistically significant. Statistical analyses were performed with IBM SPSS software (version 26.0, IBM Corp) and MedCalc Statistical Software (version 14.8.1).

## Results

### Participant characteristics

A total of 100 participants (median age: 55.0 years [IQR 45.0, 61.0 years old], range 20–75 years, 51 females) were prospectively included (Table 1). On percutaneous liver biopsy, 30 (30.0%) patients had grade 1 steatosis, 28 (28.0%) were classified as grade 2, and 42 (42.0%) had grade 3 steatosis. Fifty-one (51.0%) patients had advanced fibrosis at histopathology.

**Table 1:** Clinical, laboratory, and histopathological characteristics of the final cohort, with their comparison with steatosis grades

	Total (*n* = 100)	Steatosis grade 1 (*n* = 30)	Steatosis grade 2 (*n* = 28)	Steatosis grade 3 (*n* = 42)	*p*-value
*Clinical and laboratory data*					
**Age** (years)	55.0 (45.0, 61.0)	59.5 (52.3, 68.0)	57.0 (44.3, 61.8)	50.5 (43.3, 56.0)	**0.001**
**Sex**					0.847
Males	49 (49.0)	14 (46.7)	15 (53.6)	20 (47.6)	
Females	51 (51.0)	16 (53.3)	13 (46.4)	22 (52.4)	
**BMI** (kg/m^2^)	30.3 (27.8, 34.4)	29.5 (27.7, 31.3)	30.9 (26.7, 34.2)	32.3 (28.6, 36.7)	0.095
**BMI classification**					0.530
Normal weight	7 (7.0)	3 (10.0)	2 (7.1)	2 (4.8)	
Overweight	40 (40.0)	15 (50.0)	10 (35.7)	15 (35.7)	
Obesity	53 (53.0)	12 (40.0)	16 (57.1)	25 (59.5)	
**AST** (U/L)	34.0 (25.3, 55.5)	31.5 (22.0, 51.5)	29.0 (22.0, 41.8)	42.0 (26.8, 63.3)	**0.013**
**ALT** (U/L)	51.0 (32.3, 76.8)	44.5 (26.3, 56.5)	46.5 (26.8, 83.8)	60.5 (42.0, 97.3)	**0.006**
**GGT** (U/L)	52.5 (26.3, 94.8)	61.5 (25.5, 79.8)	36.0 (23.3, 134.0)	58.5 (28.8, 100.5)	0.489
**ALP** (U/L)	84.5 (63.3, 115.0)	87.0 (75.3, 115.8)	74.0 (56.8, 106.5)	90.5 (62.8, 124.3)	0.191
**Total bilirubin** (mg/dL)	0.6 (0.4, 1.0)	0.6 (0.5, 0.9)	0.6 (0.4, 1.0)	0.6 (0.4, 0.9)	0.352
**Hemoglobin** (g/dL)	14.4 (12.9, 15.5)	14.6 (13.3, 15.6)	13.8 (12.3, 14.8)	14.4 (13.1, 16.0)	0.152
**WBC** (× 10^3^/µL)	6.8 (5.8, 8.6)	7.4 (5.6, 8.5)	6.7 (5.7, 8.1)	6.7 (5.8, 8.7)	0.726
**Platelet count** (× 10^3^/µL)	239.0 (194.3, 282.8)	221.0 (174.8, 284.3)	267.0 (181.8, 301.8)	214.0 (208.8, 281.0)	0.459
**INR**	1.0 (1.0, 1.1)	1.0 (1.0, 1.1)	1.0 (0.9, 1.2)	1.0 (0.9, 1.1)	0.327
**Albumin** (g/dL)	4.2 (4.0, 4.5)	4.2 (3.8, 4.5)	4.1 (3.9, 4.4)	4.3 (4.0, 4.8)	0.066
**Glycaemia** (mg/dL)	98.5 (90.3, 122.8)	99.5 (90.8, 126.3)	99.0 (91.0, 119.8)	97.0 (88.8, 123.3)	0.782
**Total cholesterol** (mg/dL)	179.5 (150.3, 211.0)	179.5 (152.5, 208.8)	180.0 (150.3, 232.5)	177.0 (150.0, 203.5)	0.770
**Triglycerides** (mg/dL)	119.0 (92.0, 167.0)	121.5 (99.3, 148.3)	116.5 (88.3, 172.3)	115.0 (89.0, 175.0)	0.952
**Skin-to-liver capsule distance** (cm)	2.2 (1.9, 2.7)	2.1 (1.9, 2.5)	2.0 (1.8, 2.6)	2.5 (2.1, 2.8)	0.079
**Spleen length** (cm)	10.6 (9.4, 11.7)	10.1 (9.2, 11.2)	10.6 (9.8, 11.9)	10.8 (9.3, 11.9)	0.277
**TE** (kPa)	8.3 (5.9, 12.1)	9.3 (6.2, 11.9)	7.7 (5.7, 9.7)	7.5 (5.6, 14.1)	0.492
**CAP** (dB/m)	303.5 (261.5, 345.8)	267.0 (237.8, 323.0)	287.5 (241.5, 341.8)	331.5 (289.3, 358.8)	**0.001**
*Histopathological data*					
**Steatosis** (%)	60.0 (30.0, 70.0)	20.0 (20.0, 30.0)	50.0 (40.0, 60.0)	77.5 (70.0, 90.0)	**< 0.001**
**Inflammation**					0.385
0	3 (3.0)	1 (3.3)	0 (0)	2 (4.8)	
1	53 (53.0)	13 (43.3)	14 (50.0)	26 (61.9)	
2	43 (43.0)	16 (53.3)	14 (50.0)	13 (31.0)	
3	1 (1.0)	0 (0)	0 (0)	1 (2.4)	
**Ballooning**					**0.022**
0	39 (39.0)	5 (16.7)	15 (53.6)	19 (45.2)	
1	43 (43.0)	18 (60.0)	7 (25.0)	18 (42.9)	
2	18 (18.0)	7 (23.3)	6 (21.4)	5 (11.9)	
**NAS**	4 (4, 5)	4 (3, 4)	4 (3, 5)	5 (6, 6)	**< 0.001**
**Fibrosis stage**					0.531
F0	3 (3.0)	0 (0)	2 (7.1)	1 (2.4)	
F1	30 (30.0)	7 (23.3)	9 (32.1)	14 (33.3)	
F2	16 (16.0)	4 (13.3)	6 (21.4)	6 (14.3)	
F3	36 (36.0)	14 (46.7)	6 (21.4)	16 (38.1)	
F4	15 (15.0)	5 (16.7)	5 (17.9)	5 (11.9)	

Continuous variables are reported as median and interquartile range (25th to 75th percentile), and categorical variables are reported as numbers and percentages. Statistically significant values (*p* < 0.05) are highlighted in bold

*ALT* alanine transaminase, *AST* aspartate transaminase, *ALP* alkaline phosphate, *BMI* body mass index, *CAP* controlled attenuation parameter, *GGT* gamma-glutamyltransferase, *INR* international normalized ratio, *NAS* NAFLD activity score, *TE* transient elastography, *WBC* white blood cells

Statistically significant differences according to steatosis grades were observed in age (*p* = 0.001), aspartate transaminase (*p* = 0.013), alanine transaminase (*p* = 0.006), CAP measurements (*p* = 0.001), percentage of steatosis at histopathological analysis (*p* < 0.001), ballooning (*p* = 0.022), and NAS (*p* < 0.001).

### Diagnostic performance of UGAP

**Fig. 1:**
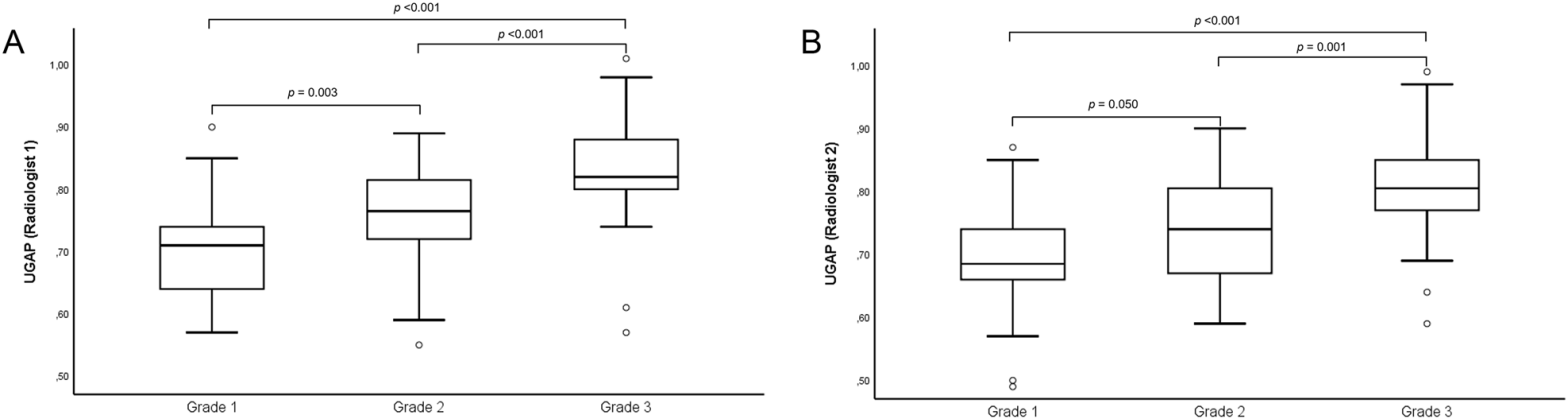
Plot box graphs with pairwise comparison of ultrasound-guided attenuation parameter (UGAP) measurements provided by the two radiologists (**A**: R1; **B**: R2) in different steatosis grades

**Fig. 2:**
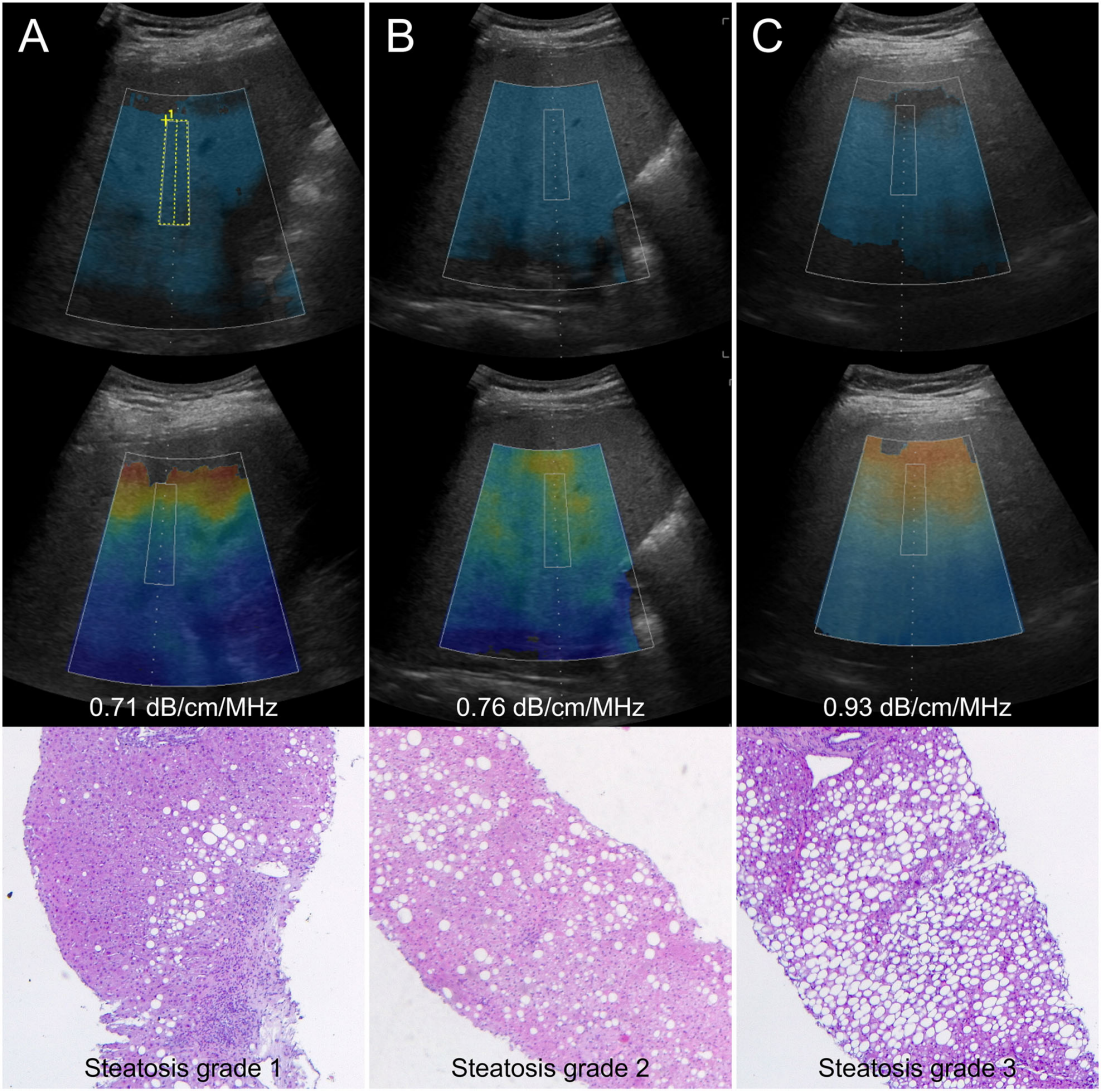
Ultrasound-guided attenuation parameter (UGAP) measurements (upper row) with the corresponding attenuation map (middle row) and histopathological correlation (lower row). **A** 68-year-old male with median UGAP of 0.71 dB/cm/MHz and 20% hepatic steatosis at histopathological analysis. **B** 41-year-old male with median UGAP of 0.76 dB/cm/MHz and 50% hepatic steatosis at histopathological analysis. **C** 21-year-old male with median UGAP of 0.93 dB/cm/MHz and 90% hepatic steatosis at histopathological analysis

The median UGAP value was 0.78 dB/cm/MHz (IQR: 0.04 dB/cm/MHz; IQR/Med: 5.55%) for R1 and 0.77 dB/cm/MHz (IQR: 0.04 dB/cm/MHz; IQR/Med: 5.50%) for R2 (Table 2). No measurement provided by both radiologists was considered invalid (all of them presented with IQR/Med being ≤ 30%, with a success rate of 100%). Median UGAP values were 0.71 dB/cm/MHz (IQR, 0.64, 0.74), 0.77 dB/cm/MHz (IQR, 0.72, 0.81), and 0.82 dB/cm/MHz (IQR, 0.80, 0.88) for R1 in patients with steatosis grade 1, 2, and 3 (*p* < 0.001), respectively, and 0.69 dB/cm/MHz (IQR, 0.66, 0.74), 0.74 dB/cm/MHz (IQR, 0.67, 0.81), and 0.81 dB/cm/MHz (IQR, 0.77, 0.86) for R2 in patients with steatosis grade 1, 2, and 3 (*p* < 0.001), respectively (Fig. 1). Examples of UGAP measurements with their histopathological correlation according to steatosis grades are provided in Fig. 2. Second measurements values provided by the first reader are reported in Supplementary Table 1.

**Table 2:** Median (Med) UGAP values (measured in dB/cm/MHz), with interquartile range (IQR) and IQR/Med (%) of the study cohort divided by steatosis grade

	Total (*n* = 100)	Steatosis grade 1 (*n* = 30)	Steatosis grade 2 (*n* = 28)	Steatosis grade 3 (*n* = 42)	*p*-value
**Median UGAP**					
Radiologist 1	0.78 (0.71, 0.84)	0.71 (0.64, 0.74)	0.77 (0.72, 0.81)	0.82 (0.80, 0.88)	**< 0.001**
Radiologist 2	0.77 (0.68, 0.82)	0.69 (0.66, 0.74)	0.74 (0.67, 0.81)	0.81 (0.77, 0.86)	**< 0.001**
**IQR**					
Radiologist 1	0.04 (0.03, 0.06)	0.05 (0.03, 0.06)	0.05 (0.03, 0.05)	0.04 (0.03, 0.06)	0.956
Radiologist 2	0.04 (0.03, 0.06)	0.04 (0.30, 0.05)	0.04 (0.03, 0.07)	0.04 (0.03, 0.06)	0.736
**IQR/Med** (%)					
Radiologist 1	5.55 (4.00, 7.50)	6.85 (3.63, 8.10)	5.85 (4.20, 7.28)	5.20 (3.70, 6.78)	0.346
Radiologist 2	5.50 (3.85, 7.65)	6.10 (4.30, 8.15)	5.40 (3.70, 8.53)	5.50 (3.80, 7.20)	0.386

Continuous variables are reported as median and interquartile range (25th to 75th percentile). Statistically significant values (*p* < 0.05) are highlighted in bold

*IQR* interquartile range, *med* median, *UGAP* ultrasound-guided attenuation parameter

A significant moderate correlation was observed between UGAP values and BMI (Table 3, *p* < 0.001 for both R1 and R2), CAP (*p* < 0.001 for both R1 and R2), percentage of steatosis at histopathological analysis (*p* < 0.001 for both R1 and R2), and NAS score (*p* < 0.001 for R1, *p* = 0.001 for R2).

**Table 3:** Correlation between UGAP measurements provided by the two radiologists and other patients’ characteristics

	Median UGAP measurements	
Characteristics	Radiologist 1	Radiologist 2
**BMI**		
ρ	0.451	0.414
*p*-value	**< 0.001**	**< 0.001**
**CAP**		
ρ	0.671	0.597
*p*-value	**< 0.001**	**< 0.001**
**Steatosis percentage**		
ρ	0.621	0.581
*p*-value	**< 0.001**	**< 0.001**
**Inflammation**		
ρ	0.043	0.001
*p*-value	0.674	0.992
**Ballooning**		
ρ	−0.072	−0.090
*p*-value	0.479	0.374
**NAS**		
ρ	0.398	0.340
*p*-value	**< 0.001**	**0.001**

Numbers represent the Spearman’s rank correlation coefficient (Spearman’s ρ) unless otherwise specified. Statistically significant values (*p* < 0.05) are highlighted in bold

*BMI* body mass index, CAP controlled attenuation parameter, NAS NAFLD activity score

Diagnostic performances of UGAP and CAP are reported in Table 4. For the diagnosis of grade ≥ 2 steatosis, the AUCs of UGAP were 0.828 (95% CI: 0.739, 0.896) for R1 and 0.779 (95% CI: 0.685, 0.856) for R2. The cutoff value of UGAP > 0.75 dB/cm/MHz was associated with a sensitivity of 67.1–75.7% and a specificity of 80.0–86.7% for the diagnosis of grade ≥ 2 steatosis. For the diagnosis of grade 3 steatosis, the AUCs of UGAP were 0.830 (95% CI: 0.742, 0.898) for R1 and 0.803 (95% CI: 0.711, 0.876) for R2. The cutoff value of UGAP > 0.75 dB/cm/MHz was associated with a sensitivity of 85.7–90.5% and a specificity of 67.2–70.7% for the diagnosis of grade 3 steatosis.

**Table 4:** Performance of UGAP and CAP for the diagnosis of grade ≥ 2 or grade 3 steatosis with sensitivity and specificity according to the optimal cutoffs

Steatosis	AUC (95% CI)	*p*-value	Cutoff	Se (95% CI)	Sp (95% CI)	TP	TN	FP	FN
**Grade** ≥ **2**									
UGAP Radiologist 1	0.828 (0.739, 0.896)	< 0.001	> 0.75	75.7 (64.0, 85.2)	86.7 (69.3, 96.2)	53	26	4	17
UGAP Radiologist 2	0.779 (0.685, 0.856)	< 0.001	> 0.75	67.1 (54.9, 74.1)	80.0 (61.4, 92.3)	47	24	6	23
CAP	0.668 (0.567, 0.759)	0.006	> 278	71.4 (59.4, 81.6)	60.0 (40.6, 77.3)	50	18	12	20
**Grade 3**									
UGAP Radiologist 1	0.830 (0.742, 0.898)	< 0.001	> 0.75	90.5 (77.4, 97.3)	67.2 (53.7, 79.0)	38	39	19	4
UGAP Radiologist 2	0.803 (0.711, 0.876)	< 0.001	> 0.75	85.7 (71.5, 94.6)	70.7 (57.3, 81.9)	36	41	17	6
CAP	0.716 (0.617, 0.802)	< 0.001	> 306	69.0 (52.9, 82.4)	70.7 (57.3, 81.9)	29	41	17	13

Cutoff values were determined according to the Youden index and they are provided in dB/cm/MHz for UGAP and in dB/m for CAP

*FN* false negative, *FP* false positive, *Se* sensitivity, Sp specificity, *TN* true negative, *TP* true positive

**Fig. 3:**
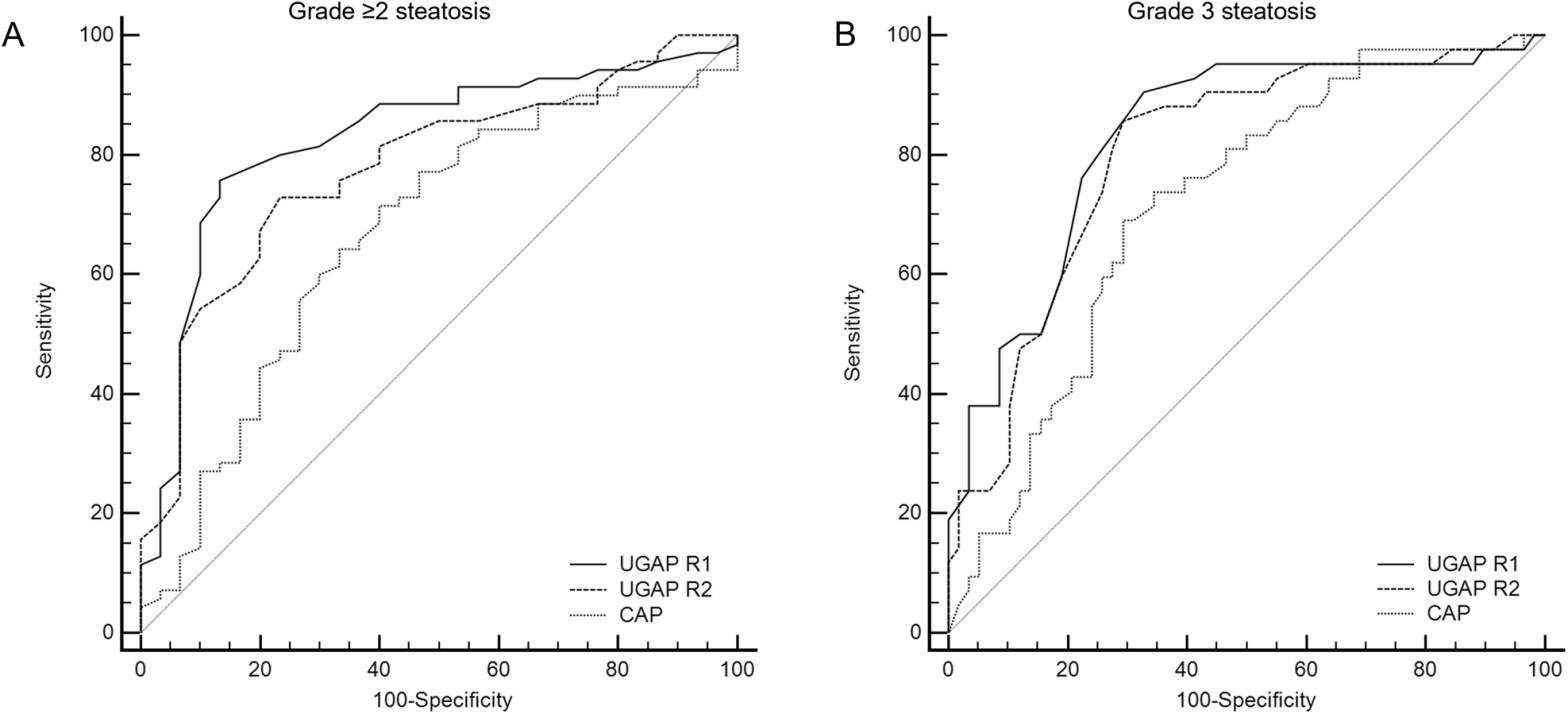
Receiver operating characteristic curves of ultrasound-guided attenuation parameter (UGAP) and controlled attenuation parameter (CAP) for the diagnosis of grade ≥ 2 (**A**) and grade 3 (**B**) steatosis

Comparison of the diagnostic performance between the two radiologists was not statistically different for grade ≥ 2 (*p* = 0.113) and grade 3 (*p* = 0.394) steatosis. The diagnostic performance of UGAP was higher compared to CAP for both the diagnosis of steatosis grade ≥ 2 (R1, *p* = 0.005; R2, *p* = 0.061) and grade 3 (R1, *p* = 0.006; R2, *p* = 0.083) steatosis. Receiver operating characteristic curves are provided in Fig. 3.

In patients with obesity, the diagnostic performance of UGAP for grade ≥ 2 steatosis was 0.796 (95% CI: 0.628, 0.963) for R1 and 0.736 (95% CI: 0.570, 0.902) for R2. In patients without obesity, the diagnostic performance of UGAP for grade ≥ 2 steatosis was 0.820 (95% CI: 0.699, 0.941) for R1 and 0.781 (95% CI: 0.651, 0.910) for R2. On multivariable analysis (Supplementary Table 2), including clinical and histopathological characteristics, only the percentage of steatosis was an independent variable associated with UGAP > 0.75 dB/cm/MHz (OR: 1.08, 95% CI: 1.05, 1,12; *p* < 0.001).

### Inter- and intra-operator agreement

The inter-operator reliability was excellent, with an ICC of 0.92 (95% CI: 0.87, 0.95). The intra-operator reliability was also excellent, with an ICC of 0.95 (95% CI: 0.92, 0.96). Bland-Altman analysis showed that the mean difference between operators was 0.01 dB/cm/MHz (95% limits of agreement, −0.09 to 0.12 dB/cm/MHz), and the mean difference between measurements of the first radiologist was 0.01 dB/cm/MHz (95% limits of agreement, −0.08 to 0.09 dB/cm/MHz).

### Diagnostic performance of 2D-SWE

**Fig. 4:**
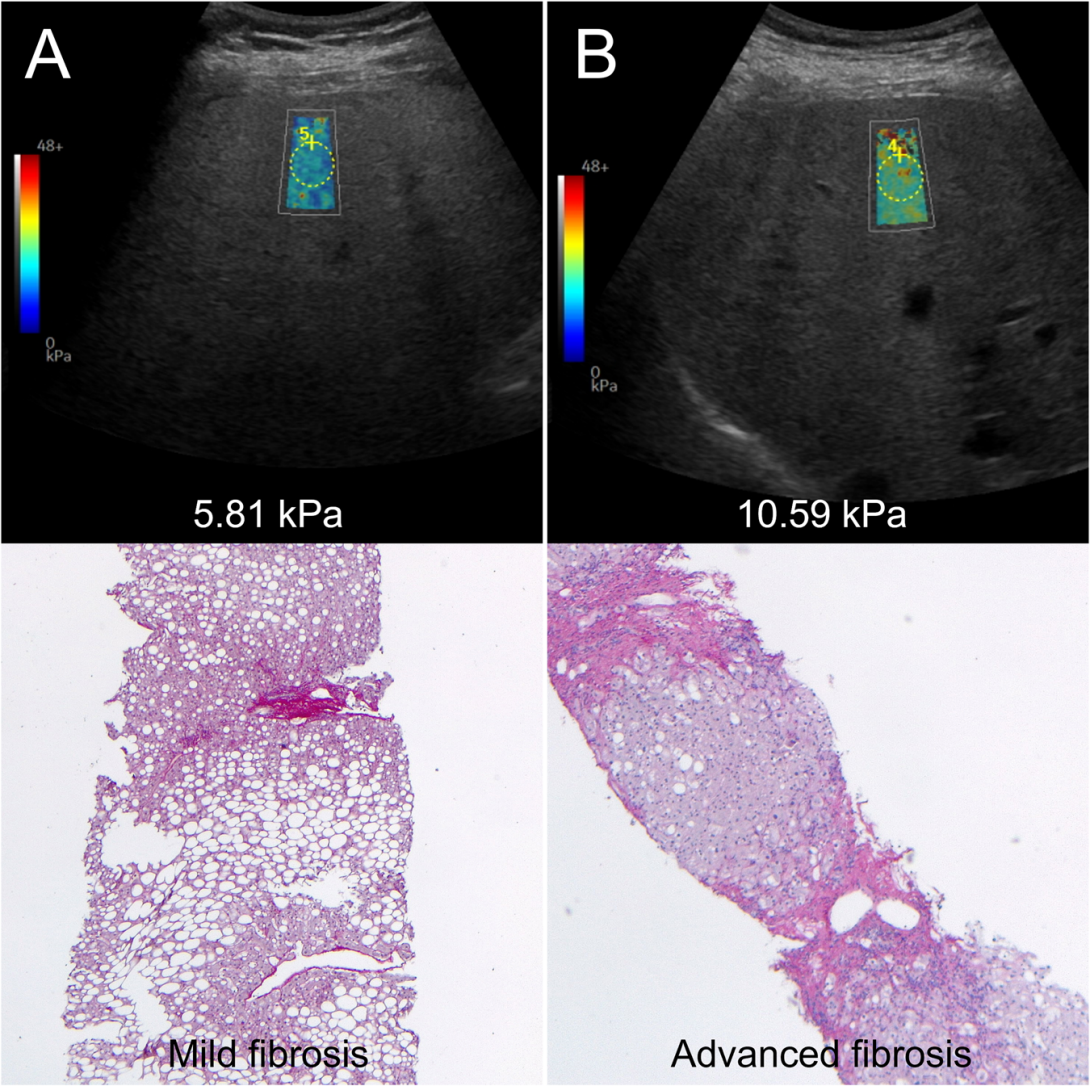
2D shear wave elastography (2D-SWE) liver stiffness measurements (upper row) with the corresponding histopathological correlation (lower row). **A** 21-year-old male with median liver stiffness measurement of 5.81 kPa and periportal fibrosis (F1c) at histopathological analysis. **B** 58-year-old female liver stiffness measurement of 10.59 kPa and periportal and bridging fibrosis with nodular transformation (F4) at histopathological analysis

The 2D-SWE was measured in 94 patients. Among them, valid (IQR/Med ≤ 30%) liver stiffness measurements were obtained in 68/94 (72.3%) patients with 2D-SWE; particularly in five patients, the twelve measurements could not be completed due to the presence of artifacts, while 21 patients had an IQR/Med > 30%. In patients with valid measurements, 43/68 (63.2%) had significant fibrosis, and 31/68 (45.6%) had advanced fibrosis. In the subgroup of patients with valid measurements, median liver stiffness was 6.75 kPa (IQR: 1.15 dB/cm/MHz; IQR/Med: 16.30%). Liver stiffness measured with 2D-SWE was significantly higher in patients with significant fibrosis compared to patients without significant fibrosis (median 7.26 [IQR: 6.19, 9.50] vs 5.57 [IQR: 4.55, 6.66]; *p* < 0.001) and in patients with advanced fibrosis compared to patients without advanced fibrosis (median 8.39 [IQR: 6.96, 10.15] vs 5.81 [IQR: 4.89, 6.80]; *p* < 0.001). Moderate correlation was observed between 2D-SWE values and fibrosis staging (ρ: 0.641, *p* < 0.001) or TE (ρ: 0.669, *p* < 0.001). Examples of 2D-SWE measurements with histopathological correlates are provided in Fig. 4.

Diagnostic performances of 2D-SWE and TE are reported in Table 5. For the diagnosis of significant fibrosis, 2D-SWE had an AUC of 0.807 (95% CI: 0.693, 0.893), and the optimal cutoff value was > 5.85 kPa, with a sensitivity of 86.0% and a specificity of 64.0%. For the diagnosis of advanced fibrosis, 2D-SWE had an AUC of 0.861 (95% CI: 0.767, 0.934), and the optimal cutoff value was > 6.75 kPa with a sensitivity of 80.6% and a specificity of 75.7%.

**Table 5:** Performance of 2D shear wave elastography (2D-SWE) and transient elastography (TE) for the diagnosis of significant (F2-F4) and advanced (F3-F4) fibrosis with sensitivity and specificity according to the optimal cutoffs

Fibrosis	AUC (95% CI)	*p*-value	Cutoff	Se (95% CI)	Sp (95% CI)	TP	TN	FP	FN
**F** ≥ **2**									
2D-SWE	0.807 (0.693, 0.893)	< 0.001	> 5.85	86.0 (72.2, 94.7)	64.0 (42.5, 82.0)	37	16	9	6
TE	0.847 (0.739, 0.922)	< 0.001	> 9.7	55.8 (39.9, 70.9)	100 (86.3, 100)	24	25	0	19
**F** ≥ **3**									
2D-SWE	0.862 (0.757, 0.934)	< 0.001	> 6.75	80.6 (62.5, 92.5)	75.7 (58.8, 88.2)	25	28	9	6
TE	0.835 (0.725, 0.914)	< 0.001	> 10.4	64.5 (45.4, 80.8)	97.3 (85.8, 99.9)	20	36	1	11

Cutoff values were determined according to the Youden index and they are provided in kPa

*FN* false negative, *FP* false positive, *Se* Sensitivity, *Sp* Specificity, *TN* true negative, TP true positive

**Fig. 5:**
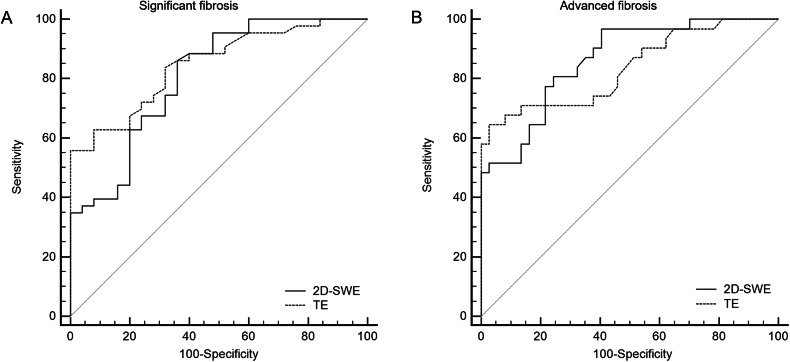
Receiver operating characteristic curves of 2D shear wave elastography (2D-SWE) and transient elastography (TE) for the diagnosis of significant (**A**) and advanced (**B**) fibrosis

Comparison of the diagnostic performance in the 68 participants with reliable 2D-SWE and TE was not statistically difference for significant (*p* = 0.482) and advanced (*p* = 0.566) fibrosis. Receiver operating characteristic curves are provided in Fig. 5.

Comparison of patients with and without valid 2D-SWE measurements is provided in Supplementary Table 3. Patients with invalid measurements were more frequently female patients (76.9% vs 39.7%, *p* = 0.001), with higher BMI (34.2 kg/m^2^ vs 29.8 kg/m^2^, *p* = 0.017) and higher skin-to-liver capsule distance (2.7 cm vs 2.1 cm, *p* < 0.001). No significant differences were observed according to steatosis grading (*p* = 0.875) or other histopathological characteristics.

## Discussion

In this study with patients with biopsy-proven MASLD, UGAP measurements provided a good performance for the noninvasive quantification of hepatic steatosis, with excellent intra- and inter-operator reproducibility. The mean UGAP values increased with the increasing steatosis grade and positively correlated with the percentage of steatosis and NAS at histopathological analysis and with BMI. The optimal cutoff of > 0.75 dB/cm/MHz provided a sensitivity of 67.1–75.7% and a specificity of 80.0–86.7% for the diagnosis of moderate-to-severe steatosis. These results can be relevant for clinical practice and noninvasive assessment of patients with MASLD. Particularly, UGAP can be easily measured with standard ultrasound equipment during the initial patient evaluation in a short examination time, it can be used to effectively stratify steatosis, and to monitor treatment changes at short-term follow-ups, due to its noninvasiveness. Notably, the performance of UGAP was higher than CAP, and all measurements acquired were considered reliable, with IQR/Med always being lower than 30%.

Few prior studies evaluated the performance of UGAP using heterogeneous reference standards for steatosis grading. In a large prospective study including patients with different etiologies of chronic liver disease, UGAP provided an excellent correlation with MRI-based fat fraction (PDFF) with an AUC of 0.912 and an optimal cutoff of 0.71 dB/cm/MHz for the diagnosis of grade ≥ 2 steatosis [14]. Kuroda et al described an AUC of 0.906 (optimal cutoff of 0.72 dB/cm/MHz) for identifying grade ≥ 2 steatosis with UGAP in 105 biopsy-proven patients with MASLD [17]. Ogino et al analyzed 84 patients with biopsy-proven MASLD and reported an AUC of 0.95 with an optimal UGAP cutoff of 0.71 dB/cm/MHz for the diagnosis of grade ≥ 2 steatosis [18]. Our study differs from these two prior ones due to the higher prevalence of obesity (53% in this study) and elevated BMI (median 30.3 kg/m^2^) in the study participants. It is likely that differences in patient characteristics and prevalence of obesity explain the slightly lower performance of UGAP in our study when compared to prior investigation. Indeed, the performance of UGAP in our study was lower in patients with obesity compared to normal or overweight patients. However, the percentage of steatosis at histopathology was the only factor independently associated with UGAP at multivariable analysis. Furthermore, in our study UGAP outperformed CAP, and this finding is consistent with prior studies [16, 17]. CAP has a higher failure rate in patients with high BMI, and measurements are obtained blindly, without ultrasound images, and they can be affected by several factors, including the skin-to-liver capsule distance and the probe type [8].

In our study, the inter- and intra-operator reliability of UGAP was excellent. A recent study by Zhao et al provided similar results for UGAP values measured by two radiologists, reporting an ICC of 0.862 [23]. Interestingly, the intra-operator reproducibility was high either if the exams were acquired on the same day or on different days in that study [23]. Furthermore, UGAP values were not affected by breathing manipulation, patient positions, or diet statuses [23]. High diagnostic performance of UGAP was also maintained with a reduced number of measurements (six vs twelve standard measurements) [19]. High reproducibility and stability in measurements are the major strengths of UGAP, which can also be acquired by operators with low experience in liver imaging.

SWE allows the noninvasive assessment of hepatic fibrosis, and it can be acquired in the same US examination, before or after UGAP. In this way, a multiparametric ultrasound evaluation of patients with MASLD can allow a comprehensive screening for focal liver lesions on B-mode images, steatosis on UGAP, and fibrosis on 2D-SWE is a single, noninvasive, rapid examination. Prior studies validated the performance of SWE in patients with MASLD [24, 25]. In a recent prospective study, Furlan et al reported no statistically significant differences between TE and 2D-SWE for the detection of significant and advanced fibrosis in patients with MASLD, with a 92% valid measurement rate with 2D-SWE [25]. In the present study, there was a high rate of 2D-SWE with invalid measurements. Factors affecting the 2D-SWE measurements are a matter of debate. A study by Kumada et al reported that the presence of severe hepatic steatosis can overestimate the liver stiffness measurements with 2D-SWE [26]. In the current study, no difference was observed in steatosis grade or percentage in patients with invalid 2D-SWE. Conversely, patients with invalid 2D-SWE measurements had significantly higher BMI and skin-to-liver capsule distance, factors that can explain the high failure rate in the current investigation.

Some limitations should be acknowledged in this single-center prospective study. The most important limitation is the lack of a control group with patients without steatosis. This was related to the fact that the biopsy was performed according to clinical indication in a patient with suspected MASLD/MASH or with abnormal liver function test and to evaluate the severity of fibrosis. This resulted in 51% of patients having advanced fibrosis, which may impact the generalizability of the results. Further studies with a control group of patients without hepatic steatosis are needed in order to confirm the diagnostic performance of UGAP in patients with a low amount of steatosis. Although liver biopsy is considered the reference standard for steatosis and fibrosis in patients with MASLD, its accuracy can be affected by the heterogeneity of the distribution of hepatic steatosis in the liver parenchyma. To mitigate potential bias, all the biopsies were performed by dedicated gastroenterologists and reviewed by the same experienced liver pathologist. Lack of data about MR elastography and MRI-PDFF further limit the interpretation of our results.

In conclusion, UGAP measurements provide a good performance for the diagnosis of moderate-to severe steatosis in patient with MASLD with and excellent inter- and intra-operator reproducibility. 2D-SWE provided good performance for fibrosis staging, although the failure rate is high in patients with high BMI and skin-to-liver capsule distance.

## Supplementary information

ELECTRONIC SUPPLEMENTARY MATERIAL

## Abbreviations

2D-SWE: Two-dimensional shear wave elastography
AUC: Area under curve
BMI: Body mass index
CAP: Controlled attenuation parameter
CI: Confidence interval
ICC: Intraclass correlation coefficient
IQR: Interquartile range
MASLD: Metabolic dysfunction-associated steatotic liver disease
NAS: NAFLD activity score
OR: Odds ratio
ROI: Region of interest
UGAP: Ultrasound-guided attenuation parameter
