# Supplementary material for: Performance of ultrasound-guided attenuation parameter and 2D shear wave elastography in patients with metabolic dysfunction-associated steatotic liver disease
Source: Eur Radiol. 2024 Oct 7;35(4):2339–50. doi: 10.1007/s00330-024-11076-w (PMC11914239; doi:10.1007/s00330-024-11076-w)
Supplement: Supplementary file 1 — ELECTRONIC SUPPLEMENTARY MATERIAL [file 330_2024_11076_MOESM1_ESM.pdf]

Performance of Ultrasound-Guided Attenuation Parameter and 2D Shear Wave Elastography in patients with metabolic dysfunction-associated steatotic liver disease

ELECTRONIC SUPPLEMENTARY MATERIAL

Supplementary Table 1: Median (Med) UGAP values (measured in dB/cm/MHz), with interquartile range (IQR) and IQR/Med (%) of the study cohort with different steatosis grade according to the second measurements of radiologist 1.

	Total ($n = 100$)	Steatosis grade 1 ($n = 30$)	Steatosis grade 2 ($n = 28$)	Steatosis grade 3 ($n = 42$)	*p* value
**Median UGAP**	0.77 (0.70, 0.82)	0.68 (0.65, 0.74)	0.76 (0.70, 0.80)	0.81 (0.77, 0.89)	**< 0.001**
**IQR**	0.04 (0.03, 0.05)	0.04 (0.03, 0.05)	0.04 (0.03, 0.06)	0.04 (0.03, 0.05)	0.705
**IQR/Med** (%)	4.82 (3.60, 6.90)	5.80 (3.95, 7.70)	5.15 (3.63, 8.68)	4.30 (3.20, 5.90)	0.081

Continuous variables are reported as median and interquartile range (25th to 75th percentile). Statistically significant values ($p < 0.05$) are highlighted in bold.
Abbreviations: IQR: Interquartile Range; med: median; UGAP: Ultrasound-Guided Attenuation Parameter.

Supplementary Table 2: Univariate and multivariate analysis of clinical, laboratory, and histopathological characteristics according to the UGAP cutoff value.

	**Univariate analysis**			**Multivariate analysis**	
	**UAGP ≤0.75** (*n* = 43)	**UGAP >0.75** (*n* = 57)	***p* value**	**OR** (95% CI)	***p* value**
Clinical and laboratory data					
**Age** (years)	58.0 (45.0, 65.0)	54.0 (46.0, 59.0)	0.105	-	
**Male sex**	19 (44.2)	30 (52.6)	0.403	-	
**BMI** (kg/m^2)	28.5 (26.7, 30.2)	32.6 (29.4, 36.5)	**< 0.001**	1.23 (1.00, 1.52)	0.053
**AST** (U/L)	34.0 (24.0, 56.0)	36.0 (24.5, 55.5)	0.689	-	
**ALT** (U/L)	47.0 (32.0, 72.0)	55.0 (38.5, 87.0)	0.173	-	
**GGT** (U/L)	62.5 (27.0, 124.0)	48.5 (25.5, 82.5)	0.442	-	
**ALP** (U/L)	87.0 (65.0, 112.0)	82.0 (62.0, 119.5)	0.369	-	
**Total bilirubin** (mg/dL)	0.7 (0.4, 1.0)	0.6 (0.4, 0.9)	0.237	-	
**Hemoglobin** (g/dL)	14.5 (12.9, 15.0)	14.3 (12.9, 15.5)	0.540	-	
**WBC** ($x10^3/\mu L$)	6.8 (5.8, 8.5)	6.7 (5.8, 8.7)	0.837	-	
**Platelet count** ($x10^3/\mu L$)	218.0 (176.0, 283.0)	244.0 (204.0, 292.5)	0.210	-	
**INR**	1.0 (1.0, 1.1)	1.0 (1.0, 1.1)	0.174	-	
**Albumin** (g/dL)	4.2 (3.8, 4.5)	4.2 (4.0, 4.6)	0.270	-	
**Glycaemia** (mg/dL)	97.0 (90.0, 123.0)	100.0 (90.5, 122.8)	0.728	-	
**Total cholesterol** (mg/dL)	180.0 (154.0, 211.0)	179.0 (150.0, 211.8)	0.730	-	
**Triglycerides** (mg/dL)	112.0 (97.0, 137.0)	120.5 (87.3, 172.3)	0.601	-	
**Skin-to-liver capsule distance** (cm)	2.1 (1.7, 2.5)	2.5 (2.1, 2.9)	**< 0.001**	2.62 (0.56, 12.26)	0.221
**Spleen length** (cm)	10.2 (9.4, 11.3)	10.8 (9.5, 11.9)	0.218	-	
**TE** (kPa)	8.0 (5.5, 10.7)	9.2 (6.0, 13.8)	0.165	-	
Histopathological data					
**Steatosis** (%)	30.0 (20.0, 50.0)	70.0 (60.0, 80.0)	**< 0.001**	1.08 (1.05, 1,12)	**< 0.001**
**Inflammation** (2-3)	19 (44.2)	25 (43.9)	0.974	-	
**Ballooning** (1-2)	30 (69.8)	31 (54.4)	0.118	-	
**NAS**	4 (3, 4)	5 (4, 8)	**< 0.001**	1.41 (0.72, 2.76)	0.314
**Advanced fibrosis** (F3-F4)	21 (48.8)	30 (52.6)	0.707	-	

UGAP measurements were based on the radiologists 1. Continuous variables are reported as median and interquartile range (25th to 75th percentile), categorical variables are reported as

numbers and percentages. Statistically significant values ($p < 0.05$) are highlighted in bold. Statistically significant variables at univariate analysis were included in the multivariate logistic regression analysis.

Abbreviations: ALT: Alanine Transaminase, AST: Aspartate Transaminase; ALP: Alkaline Phosphate; BMI: Body Mass Index; CAP: Controlled Attenuation Parameter; CI: Confidence Interval; GGT: gamma-glutamyltransferase; INR: International Normalized Ratio; OR: Odds Ratio; NAS: NAFLD Activity Score; TE: Transient Elastography; WBC: White Blood Cells.

Supplementary Table 3: Comparison of clinical and histopathological characteristics in patients with and without valid 2D-SWE measurements (n = 94).

	Valid measurements (n = 68)	Invalid measurements (n = 26)	p value
**Age** (years)	54.5 (41.8, 60.8)	56.0 (48.8, 61.3)	0.424
**Sex**			**0.001**
Males	41 (60.3)	6 (23.1)	
Females	27 (39.7)	20 (76.9)	
**BMI** (kg/m^2)	29.8 (27.5, 32.7)	34.2 (28.8, 36.8)	**0.017**
**BMI classification**			0.264
Normal weight	6 (8.8)	1 (3.8)	
Overweight	30 (44.1)	8 (30.8)	
Obesity	32 (47.1)	17 (65.4)	
**TE** (kPa)	7.45 (5.92, 11.88)	9.40 (5.98, 17.0)	0.202
**CAP** (dB/m)	219.5 (251.3, 347.8)	297.5 (261.5, 334.8)	0.886
**Skin-to-liver capsule distance** (cm)	2.1 (1.9, 2.5)	2.7 (2.2, 3.0)	**<0.001**
**UGAP** (dB/cm/MHz)	0.77 (0.71, 0.83)	0.79 (0.74, 0.85)	0.310
**Steatosis grade**			0.954
1	20 (29.4)	7 (26.9)	
2	19 (27.9)	8 (30.8)	
3	29 (42.6)	11 (42.3)	
**Steatosis** (%)	60.0 (30.0, 70.0)	60.0 (30.0, 70.0)	0.875
**Inflammation**			0.517
0	3 (4.4)	0 (0)	
1	38 (55.9)	13 (50.0)	
2	26 (38.2)	13 (50.0)	
3	1 (1.5)	0 (0)	
**Ballooning**			0.477
0	30 (44.1)	8 (30.8)	
1	26 (38.2)	13 (50.0)	
2	12 (17.6)	5 (19.2)	
**NAS**	4.0 (3.0, 5.0)	4.5 (4.0, 5.0)	0.161
**Fibrosis stage**			0.262
F0	3 (4.4)	0 (0)	
F1	22 (32.4)	7 (26.9)	
F2	12 (17.6)	3 (11.5)	
F3	24 (35.3)	9 (34.6)	
F4	7 (10.3)	7 (26.9)	

Continuous variables are reported as median and interquartile range (25th to 75th percentile), categorical variables are reported as numbers and percentages. Statistically significant values (p < 0.05) are highlighted in bold.

Abbreviations: BMI: Body Mass Index; NAS: NAFLD Activity Score; UGAP: Ultrasound-Guided Attenuation Parameter.
